# Great Himalayan Leaf-Nosed Bats Produce Different Territorial Calls to Respond to Sympatric Species and Non-Living Objects

**DOI:** 10.3390/ani10112040

**Published:** 2020-11-04

**Authors:** Hexuan Qin, Lei Feng, Xin Zhao, Congnan Sun, Jiang Feng, Tinglei Jiang

**Affiliations:** 1 Jilin Provincial Key Laboratory of Animal Resource Conservation and Utilization, Northeast Normal University, 2555 Jingyue Street, Changchun 130117, China; qinhx618@nenu.edu.cn (H.Q.); fengl111@nenu.edu.cn (L.F.); suncn622@nenu.edu.cn (C.S.); 2 Key Laboratory of Vegetation Ecology of Education Ministry, Institute of Grassland Science, Northeast Normal University, 2555 Jingyue Street, Changchun 130117, China; 3 School of Psychology, Inner Mongolia Normal University, Hohhot 150100, China; zhaox111@nenu.edu.cn; 4 College of Life Science, Jilin Agricultural University, 2888 Xincheng Street, Changchun 130118, China

**Keywords:** great Himalayan leaf-nosed bat, roost territory, sympatric species, non-living objects, territorial calls

## Abstract

**Simple Summary:**

Many animals produce keep-out signals to keep intruders from entering their territories. Studies have shown that bats produce territorial calls to defend the conspecifics intrusion. However, it remains unknown whether bats can adjust their territorial calls in response to different types of intruders, such as heterospecifics or non-living objects. We simulated the process of territory defense in male great Himalayan leaf-nosed bats toward two sympatric species and four non-living objects to investigate their acoustic responses. Bats displayed different acoustic responses for different types of intruders, suggesting that territorial calls of male great Himalayan leaf-nosed bats may convey emotional state information when the bats respond to invasion by sympatric species or non-living objects. Our results are valuable for understanding animal cognition and interactions among bat species from an acoustic perspective.

**Abstract:**

Territorial signals are important for reducing the cost of territory defense. Normally, male animals will produce keep-out signals to repel intruders from entering their territory. However, there is currently no evidence that bats can adjust their territorial calls to respond differently to sympatric species or non-living objects. In this study, we simulated the process of territory defense in male Great Himalayan leaf-nosed bats (*Hipposideros armiger*) toward two sympatric species (*Hipposideros pratti* and *Rhinolophus sinicus*) and four different non-living objects (a fur specimen of *H. armiger*, a bat model, a speaker, and a speaker with playback of *H. armiger* echolocation calls) to investigate their acoustic responses. There were significant differences in the territorial call complexity, syllable rate, and syllable ratio produced by *H. armiger* under the different experimental conditions. Our results confirmed that bats can adjust their territorial calls to respond to different sympatric species and non-living objects. The results will further our understanding of animal cognition and interactions among bat species from an acoustic perspective.

## 1. Introduction

A territory is defined as an area that animals exclusively occupy and actively defend [1]. Many animals such as bats, starlings, sparrows, and domestic pigeons own and defend a private territory in order to obtain better rest (including sleep) or shelter [2]. Rest and sleep occupy most of the daily life of animals relative to other activities. The quality of roost territory can affect the quality of rest, which indirectly affects the individual’s physiological state, immunity, metabolism, and cognitive memory. Ultimately high-quality rest is helpful for maximizing survival and reproductive potential [3,4].

Acoustic signals are widely used to defend against possible intruders during territorial conflicts. Among animals, it is common to distinguish intruders and neighbors through territorial calls [5]. In frogs, fish, birds, and mammals, receivers change their behavioral responses according to whether they hear calls from neighbors within their territory, neighbors outside their territory, strangers (belonging to the same species, or other species) [5,6].

Bats are one of the most highly social mammals. They normally occupy dark environments and therefore depend heavily on acoustic signals. Bats use two types of acoustic signals to communicate, echolocation, and social calls [7,8,9,10]. Echolocation is mainly used for navigation and the detection, classification, and location of specific targets, especially prey [11,12]. Echolocation calls can also encode information about the activities of vocal bats, such as obtaining food, or specific information about individuals, such as sex, age, or species identity information [9,13,14]. In contrast to echolocation calls, social calls have complex spectral characteristics that encode individual information, an attribute that is helpful for individual recognition in large groups [15,16,17,18]. For example, in Pallid bats (*Antrozous pallidus*), Spix’s disc-winged bats (*Thyroptera tricolor*), and Vampire bats (*Desmodus* spp.), social calls encode individual characteristics and group identity information [16,17,19,20]. Additionally, the social calls of bats encode various types of information during territory defense. For example, male greater white-lined bats (*Saccopteryx bilineata*) emit territorial calls that can attract females [21]. Species-specific social calls during territory defense have been observed in male pipistrelle (*Pipistrellus pipistrellus*) and male African banana bats (*Pipistrellus nanus*) [22,23]. Male *S. bilineata* produce courtship vocalizations when hearing female echolocation calls, and aggressive vocalizations when hearing male echolocation calls [24]. However, to date, no studies have focused on how bats might adjust their social calls to respond to sympatric species during territorial defense.

The great Himalayan leaf-nosed bat (*Hipposideros armiger*) mainly roosts in caves, and often coexists and roosts with several bat species such as Pratt’s leaf-nosed bat (*Hipposideros pratti*) and the Chinese horseshoe bat (*Rhinolophus sinicus*) (long personal observation by Tinglei Jiang). *H. armiger* is an ideal study species for examining the acoustic responses used for roosting territorial defense against sympatric species and non-living objects for the following reasons. First, the social calls in *H. armiger* are very complex and diverse, with 18 simple syllables, 17 complex syllables, and additional complex calls [25]. Second, *H. armiger* individuals usually maintain a distance of 10–15 cm between one another when sharing a day roost. Once this distance is encroached upon, the individuals will engage in territorial conflict [26]. Third, we previously observed in the field that when sympatric bat species fly close to an *H. armiger* roost territory, they mostly emit stepped upward frequency modulation (sUFM) calls in response to the intruders. Fourth, we previously observed in the lab that when non-living objects approach the roost of *H. armiger*, they also emitted social calls. Therefore, our experiments included sympatric species and non-living objects to investigate the differences in acoustic response to any intrusion.

In this study, we recorded the acoustic responses of *H. armiger* to intrusion by simulating the process of these intruders approaching the roost territory. We hypothesized that *H. armiger* could produce different territorial calls in response to different types of intruders during its roost territory defense. To test this hypothesis, we made the following two predictions: (1) significant differences in the territorial calls of *H. armiger* will be observed in response to conspecifics and different sympatric species; (2) significant differences in the territorial calls of *H. armiger* will be observed in response to conspecifics and non-living objects.

## 2. Materials and Methods

### 2.1. Bat Collection and Husbandry

We collected 12 adult male bats (10 *H. armiger*, 1 *H. pratti*, and 1 *R. sinicus*) from Yu cave (107°01′35.72″ E, 32°51′05.25″ N) in Hanzhong, Shanxi Province, China in May 2019. Bats were captured using mist nets and held in cloth bags until being transferred to a temporary feeding room (5 m × 3 m × 3 m) near their roost. To identify individuals, an aluminum alloy band (Porzana Ltd., East Sussex, UK; 4.2 mm, 0.12 g) was worn on the left or right forearm of the bat. The aluminum alloy bands weighed less than 0.28% of the bat’s body weight. We ensured that the bands could slide freely along the bat’s forearm without cutting into the forearm. According to our previous study [27], aluminum alloy bands do not adversely impact the behavior of bats. In order to prevent heterospecific communication prior to the experiments, the three bat species were put into three breeding cages built with wire mesh (1 m × 1 m × 1.5 m, 1.5-cm mesh diameter), and external sound-absorbing foam. Bats were fed beetle mealworms (*Tenebrio molitor*) with enriched vitamins and provide access to mineral water. The light cycle, temperature and humidity in the temporary feeding room were similar to those of the natural roost of the bats (lights on: 05:00–19:00; lights off: 19:00–05:00, temperature 18–23 °C, relative humidity 70–90%).

### 2.2. Territory Defense Experiment Assessing H. Armiger Responses to Sympatric Species

**Figure 1:**
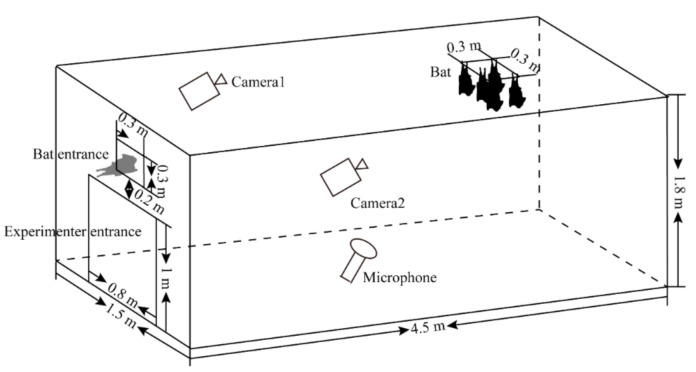
A schematic diagram of the territory defense experiment using sympatric species of *Hipposideros armiger*. The intrusive bat is shown in grey to distinguish it from roosting bats.

It is difficult to identify the sound-producing individuals based on video analysis when there are more than five individuals per cage. Therefore, for this experiment, nine *H. armiger* were randomly divided into two groups, five in one group, and four in the second group, A tenth individual was kept separate to act as an intruder. An experimental cage was built using wire mesh sound-absorbing foam to house bats during the experiments (Figure 1). To ensure that the bats roosted in a standardized location, a plastic film was affixed to the inner top edge of the experimental cage. At least 10 cm was maintained between all individuals. A bat entrance was made on the side of the experimental cage, about 3.2 m away from the roost. Additionally, an experimenter entrance was made on the same side as the bat entrance.

Territorial calls were recorded using an ultrasonic recorder Avisoft UltraSoundGate 116H (Avisoft Bioacoustics, Berlin, Germany) connected to an ultrasonic microphone (CM16/CMPA, frequency range 10 KHz ± 3 db–200 KHz ± 3 db, Avisoft Bioacoustics, Berlin, Germany) with a sampling rate of 375 kHz and 16-bit resolution. The microphone tripod was placed at an angle of 60° on the ground to face the bat’s head at a distance 1.5 m from the bat’s head. Simultaneously, we used two infrared cameras (HDR-CX 760E; Sony Corp., Tokyo, Japan) to record the bats’ behavior, one placed 1 m away from the entrance, and the other 1.5 m away from the bats. Both cameras faced the bats and were placed 1.4 m above the ground. During the experiments, we randomly placed a group of *H. armiger* into the experimental cage. After the bats had adjusted to the environment and calmed down (no echolocation calls for 1 min via observing a display outside the cage), the intrusion trials were carried out by releasing *H. armiger*, *H. pratti,* or *R. sinicus* one at a time into the bat entrance (Figure 1). To prevent *H. armiger* from becoming habituated, we released a different invasive species during each successive trial. In each experiment, we released the invading bat only after the roosting bats had habituated. The experiments were performed from 10 p.m. to 3 a.m. If the intruding bat did not fly to the roost area or the recording quality was poor, we repeated the trial until a high-quality call was obtained. In total, 255 successful trials were completed using conspecific intruders, 234 using *H. pratti* and 301 using *R. sinicus*.

### 2.3. Territory Defense Experiment Assessing H. Armiger Responses Non-Living Objects

In this study, four non-living objects (a fur specimen of *H. armiger*, a plastic bat model, an ultrasonic speaker (Ultrasonic Dynamic speaker Vifa; Avisoft Bioacoustics, Berlin, Germany), and an ultrasonic speaker playing *H. armiger* echolocation calls) were used to test the territorial responses of *H. armiger*. The fur specimens were sourced from the Northeast Normal University, and the bat model was made of plastic (length × width× depth: 0.13 m × 0.35 m × 0.03 m). Echolocation calls for the playback speaker were recorded using the same ultrasonic microphone and specifications used to record territorial calls. Specifically, the calls of one intruding *H. armiger* were recorded as it flew toward the roost position from the bat entrance of the experimental cage. The playback sound was normalized to 0.75 V with a 1.53 s duration and 22 calls/s rate, similar to the period of the pendulum used in the experiment as described below.

**Figure 2:**
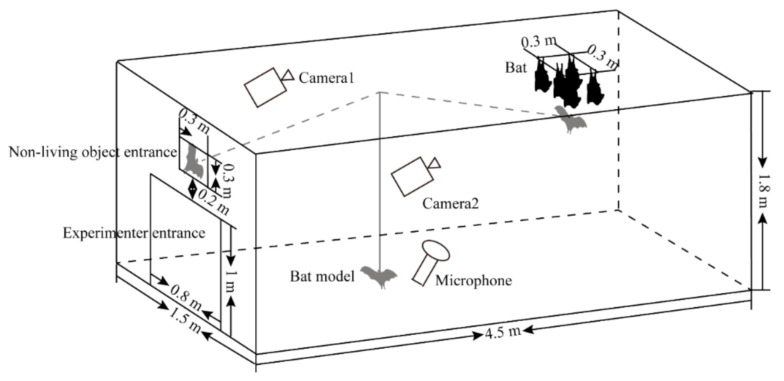
A schematic diagram of the territory defense experiment using intrusive non-living objects and roosting *Hipposideros armiger*. The intrusive object is shown in grey to distinguish it from roosting bats.

We used a single pendulum to control the different non-living objects used to invade the roost of *H. armiger*. A 1.6 m cotton string was tied 1.6 m away from the entrance. Non-living objects were tied to the string to swing them from the bat entrance to the bat roost position (Figure 2). The location was the same as those in the sympatric species territory defense experiment (Figure 2). The playback speaker was not facing the microphone when used as the invading object. The calls produced by the playback speaker were therefore not recorded. Even if the microphone recorded the calls played by the speaker, the calls were weak. This enabled them to be distinguished from the territorial calls produced by roosting bats during subsequent analysis. To prevent *H. armiger* from becoming habituated to the intrusion of particular objects, we employed a different non-living object during successive trials. Each trial was performed only after the roosting bats had habituated. The experiments were conducted from 10 p.m. to 3 a.m. If the intruding object did not reach the roost area or the recording quality was not good, we will repeat the release of the invasive object to get a high-quality call. In total, 229 successful trials were completed using the fur specimen of *H. armiger*, 352 using the bat model, 304 using the speaker, and 364 using the speaker with *H. armiger* echolocation call playback.

### 2.4. Analysis of Social Calls

We conducted a frame-by-frame video analysis to determine which individuals were vocal during each trial using a QvodPlayer (25 frames/s; Version 5.0.80, Shenzhen Qvod technology Co., Ltd., Guangdong, China). Normally only one or two individuals produced territorial calls during each trial. For analysis, we selected territorial calls from the individual with the highest-quality vocalization (good signal-to-noise ratios) and the strongest response behaviors (e.g., nodding and body movement). We classified syllables based on the visual spectrogram inspection methods of Kanwal et al. [28] and Lin et al. [25]. A syllable refers to the smallest independent unit of a vocalization surrounded by silence [28]. A call was defined as the simplest overall vocalization in the context of behavior that consisted of multiple syllables [29]. In the process of territory defense, *H. armiger* emitted territorial calls consisting of several syllable types, including sUFM, single humped frequency modulation (sHFM), plateaued paraboloid frequency modulation-single humped frequency modulation (pPFM-sHFM), and bent upward frequency modulation-single humped frequency modulation (bUFM-sHFM). Territorial calls composed of repeated sUFM were the most common, accounting for 83.52 ± 16.76% of all syllable types. We calculated the number of syllables of each call divided by the duration of the call to obtain the syllable rate for each individual. We used Avisoft-SASLab Pro (version 5.2; Avisoft Bioacoustics, Berlin, Germany) to measure the acoustic parameters of sUFMs having good signal-to-noise ratio. The averages of 12 territory calls from each individual in each experimental condition were selected, and only one high-quality sUFM syllable was selected for each call (signal-to-noise ratio >40 dB). The signal was high-pass filtered at 1 kHz and normalized to 0.75 V. We measured one temporal parameter and 29 spectrum parameters (refer Table S1 for the definitions of all acoustic parameters). Acoustic parameters were measured from spectrograms using a fast Fourier transform (FFT) length of 1024 points (Hamming window: 75% frame size and 93.75% overlap), resulting in a temporal resolution of 0.256 ms and a frequency resolution of 244 Hz.

### 2.5. Analysis of Call Complexity

To quantify the amount of information contained in the territorial calls of *H. armiger*, we calculated the call complexity, which was related to the number and diversity of the call repertoire. According to the composition of syllables in a call, we classified them into three “syllable assembly patterns”, including a single syllable in a call (i.e., sUFM), repeated syllables in a call (i.e., sUFM-sUFM), and combined syllables in a call (i.e., sUFM-bUFM-sHFM), and described the frequencies of these patterns. We then used the Shannon–Weaver diversity index (DI) to calculate call complexity [30,31]:

(1) $$DI\;=\;\frac{H_{i}}{H_{i}\;\max}\;=\;\frac{-\sum_{i\;=\;1}^{n}P_{i}\times \log_{2}\left(P_{i}\right)}{\log_{2}\left(n\right)}$$

where, *H*_i_ max is the value when all utterance frequencies were the same, *H*_i_ is the diversity index calculated based on the actual frequency, *P*_i_ is the probability of each pattern, and *n* is the number of “syllable assembly patterns”. We calculated the DI value for each individual under each experimental condition.

### 2.6. Statistical Analysis

For each individual, we analyzed the average values of acoustic parameters to avoid pseudoreplication. Additionally, several outliers were excluded during the statistical analysis. First, we used Kolmogorov–Smirnov tests to examine the normality of the data before conducting statistical analyses on calls and syllables (all *p* > 0.05). For the call analysis, all syllable types and numbers were counted, and DI was calculated (Figure S1). Differences in the syllable rate and DI were evaluated among the different experimental conditions using a one-way analysis of variance (ANOVA) and a post-hoc test (Tukey’s) using the package “multcomp” [32] in R 4.0.1 [33]. For the syllable analysis, we only took into account sUFM calls. We tested differences in the proportions of sUFM syllables among the different experimental conditions using a one-way ANOVA and a post-hoc test (Tukey’s) using the package “multcomp” [32]. We performed principal component analysis with the 30 syllable parameters of sUFM using the package “psych” [34]. The package “MASS” [35] was used to conduct linear discriminant analysis on the results of the principal component analysis. All analyses were performed in R 4.0.1 [33]. Results were expressed as mean ± standard deviation and significance was defined at the *p* < 0.05 level.

### 2.7. Ethical Statements

All procedures were in compliance with the National Natural Science Foundation of China for experiments involving vertebrate animals, and were approved by the Northeast Animal Research Authority of Northeast Normal University, China (approval number: NENU-W-2019-101). Bats were captured using mist nets and hand-draft nets, and then put into cloth bags and sent to temporary experimental stations near their roost. No bats were harmed during the capture, transport, and experimentation. We removed the aluminum alloy bands used to uniquely identify bats before they were released. After the experiment, all the captured bats were healthy and were released at the location where they were originally captured.

## 3. Results

There were a total of 2039 territorial calls produced by the nine *H. armiger* included in this study that were selected for analysis. The average number of territorial calls produced was 28.33 ± 8.63 in response to conspecifics. 26 ± 8.44 in response to *H. pratti*, and 33.44 ± 10.60 in response to *R. sinicus*. The average number of territorial calls produced was 33.78 ± 12.11 in response to the speaker, versus 40.44 ± 20.52 in response to the speaker with playback. In response to the *H. armiger* fur specimen, the average number of calls was 25.44 ± 8.55, and in response to the bat model, the average was 39.11 ± 20.04. A total of 3872 syllables were counted, and sUFM accounted for 83.52 ± 16.76% of all syllables.

### 3.1. Comparison of H. Armiger Territorial Calls in Response to Sympatric Species

**Figure 3:**
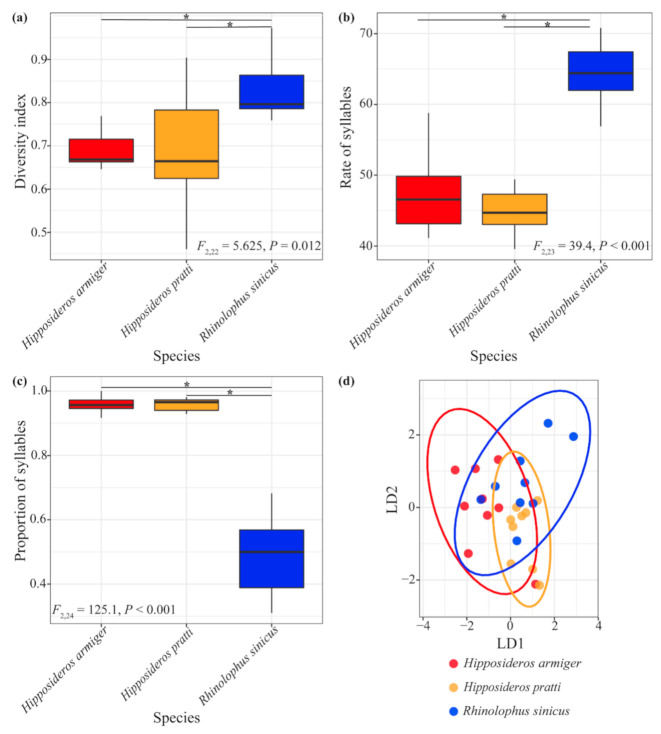
Differences in territorial calls of *Hipposideros armiger* in response to invasion by sympatric bat species. (**a**) Diversity index of territorial calls; (**b**) syllable rate of territorial calls; (**c**) proportion of territorial calls that were stepped upward frequency modulation (sUFM) syllables; (**d**) ellipses showing the 95% confidence interval were obtained from a discriminant function analysis of two principal component factor scores measured from sUFM territorial calls. Colors show groupings according to species identity (*n* = 3). * *p* < 0.05. LD1, linear discriminant function 1; LD2, linear discriminant function 2.

Our results showed that the DIs of *H. armiger* territorial calls were significantly different in response to *H. pratti* and *R. sinicus* (*F*_2,22_ = 5.625, *p* = 0.012). The DIs of territorial calls in *H. armiger* responding to both conspecifics and *H. pratti* were significantly lower than in response to *R. sinicus* (*p* < 0.05; Figure 3a). There was a significant difference in the syllable rate of territorial calls produced by *H. armiger* in response to conspecifics and the sympatric species (*F*_2,23_ = 39.4, *p* < 0.001; Figure 3b). The syllable rates of territorial calls when *H. armiger* responded to conspecifics and *H. pratti* were significantly lower than in response to *R. sinicus* (*p* < 0.001; Figure 3b). The proportion of sUFM syllables was significantly different when *H. armiger* responded to the two sympatric species (*F*_2,24_ = 125.1, *p* < 0.001), and *H. armiger* produced a higher proportion of sUFM syllables when they responded to intrusion by conspecifics and *H. pratti* than *R. sinicus* (*p* < 0.001; Figure 3c). Territorial calls of *H. armiger* responding to conspecifics and the two sympatric species could be statistically distinguished based on the acoustic parameters of sUFM syllables (Figure 3d). The 74% classification success was significantly higher than a random probability (33.3%; *p* < 0.001; Figure 3d).

### 3.2. Comparison of H. Armiger Territorial Calls in Response to Non-Living Objects

**Figure 4:**
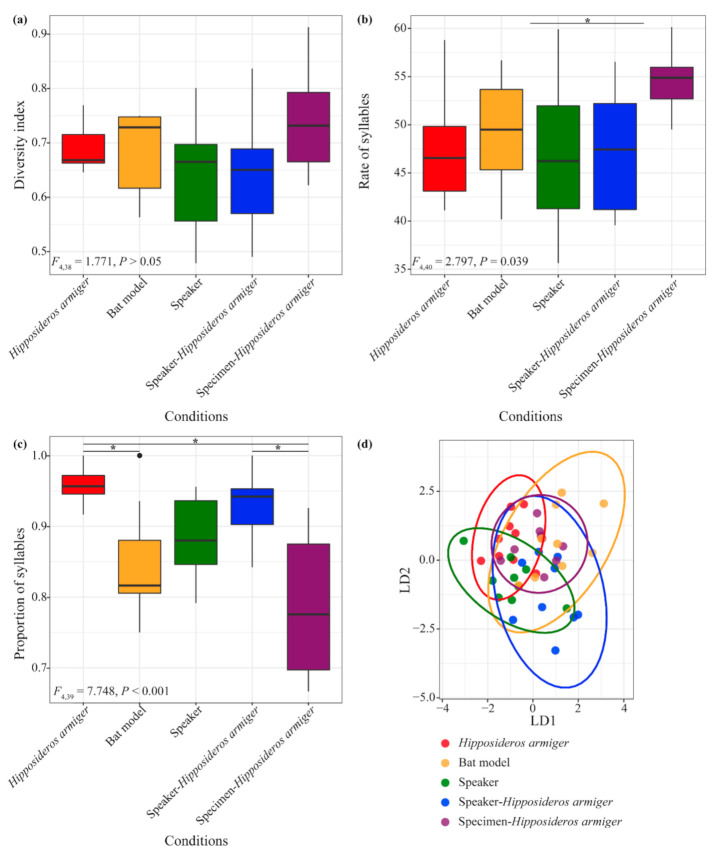
Differences in territorial calls of *Hipposideros armiger* in response to invasion by non-living objects. (**a**) Diversity index of territorial calls; (**b**) syllable rate of territorial calls; (**c**) proportion of territorial calls that were sUFM syllables; (**d**) ellipses showing the 95% confidence intervals that were obtained from a discriminant function analysis of two principal component factor scores measured from sUFM territorial calls. Colors show grouping according to object identity (*n* = 5). * *p* < 0.05. LD1, linear discriminant function 1; LD2, linear discriminant function 2.

We found no significant differences in DIs of the territorial calls when *H. armiger* responded to conspecifics versus the four different non-living objects (*F*_4,38_ = 1.771, *p* > 0.05, Figure 4a). The syllable rate of territorial calls was significantly different when *H. armiger* responded to the non-living objects (*F*_4,40_ = 2.797, *p* = 0.039). The syllable rate of territorial calls in response to the speaker was significantly greater than that in response to the fur specimen (*p* = 0.037; Figure 4b). The proportions of sUFM syllables in territorial calls when *H. armiger* responded to the non-living objects were significantly different (*F*_4,39_ = 7.748, *p* < 0.001). The proportion of sUFM syllables produced in response to conspecifics was higher than in response to the bat model (*p* = 0.01) and the fur specimen (*p* < 0.001; Figure 4c). Moreover, the proportion of sUFM syllables produced in response to the speaker with playback of *H. armiger* echolocation calls was higher than in response to the fur specimen (*p* < 0.05; Figure 4c). Territorial calls of *H. armiger* responding to conspecifics and the four non-living objects could be statistically distinguished based on the acoustic parameters of sUFM syllables (Figure 3d). The 60% classification success was significantly higher than a random probability (20%; *p* < 0.001; Figure 4d).

## 4. Discussion

In this study, we found that DIs, syllable rates, and syllable ratios of territorial calls were significantly different when *H. armiger* responded to conspecifics and two sympatric species, and the observed classification success among the three bat species was significantly higher than expected from random chance, supporting our first prediction. Moreover, there were significant differences in some parameters of territorial calls when *H. armiger* responded to conspecifics versus four non-living objects, and the observed classification success among *H. armiger* and the four non-living objects was significantly higher than random chance, supporting our second prediction. These results clearly indicate that *H. armiger* can adjust its territorial calls in response to intrusion by different sympatric species and non-living objects, suggesting that territorial calls may contain different types of information.

Big brown bats (*Eptesicus fuscus*) express their emotional state by using context-specific syllable types or by varying their temporal call structure (such as the syllable repetition rate and the number of syllables per call) [29]. In this study, the acoustic responses of *H. armiger* to conspecifics and *H. pratti* were similar, but were significantly different toward *R. sinicus*. This may be due to the fact that *H. armiger* and *H. pratti* belong to the same genus and have similar body sizes (forearm length: 95 ± 15 mm in *H. armiger* vs. 87 ± 7 mm in *H. pratti*) [36]. However, their body sizes are quite different than that of *R. sinicus* (47 ± 6 mm) [36]. The DIs of territorial calls in response to conspecifics and *H. pratti* were significantly lower than in response to *R. sinicus*, but the opposite pattern was observed for the proportions of sUFM syllables contained in territorial calls (Figure 3). This may be because *H. armiger* produced more sHFM syllables in response to *R. sinicus* (36% of all syllables) than to *H. armiger* (1.14% of all syllables) and *H. pratti* (1.58% of all syllables), leading to a greater diversity of territorial calls. These results suggest that *H. armiger* may have adjusted territorial calls to include more information in responses to distantly related species. Studies have shown that greater false vampire bats (*Megaderma lyra*) changed their call structure and increased the syllable rate according to the intensity of agonistic interactions [37]. In this study, the syllable rate of *H. armiger* territorial calls in response to conspecifics and *H. pratti* was significantly lower than that toward *R. sinicus*, which may have been due to the following two factors. First, *H. armiger* emitted more sHFM syllables when they responded to *R. sinicus*. Since the duration of sHFM was shorter than that of sUFM and other syllables, this would lead to the syllable rate being higher when responding to *R. sinicus*. Second, *H. armiger* and *H. pratti* normally roost together in the same area of the same cave, which leads to the two species being familiar with each other. However, *R. sinicus* usually does not roost together with *H. armiger* and *H. pratti*, preferring to inhabit different areas of the same cave (long personal observation by Tinglei Jiang). Therefore, differences in familiarity with *R. sinicus* may result in a higher syllable rate toward *R. sinicus* that may indicate a more aggressive state. However, further experimental examination will be required to help answer this question.

*H. armiger* could discriminate between conspecifics and the four non-living objects based on sUFM territorial calls (Figure 4d), suggesting there were differences in acoustic responses to different non-living objects. Our results showed that syllable rate was only significantly different when *H. armiger* responded to intrusion by a speaker and an *H. armiger* fur specimen. This may be because a fur specimen was much closer in appearance to a living bat than the speaker, leading to more frequent vocalizations. Additionally, the proportion of sUFM syllables when *H. armiger* responded to a conspecific was significantly higher than to the bat model and the fur specimen. Similarly, the proportion of sUFM syllables was higher when *H. armiger* responded to the speaker with playback of *H. armiger* echolocation calls than to the fur specimen. Although the bat model and the specimen were shaped like bats, the speaker with echolocation calls may have appeared more like a living bat to *H. armiger*. In this case, it is reasonable that *H. armiger* produced more frequent sUFM territorial calls to defend themselves because living bats pose a greater threat than models or specimens. On the whole, these results suggest that *H. armiger* can have different acoustic responses to living bats and non-living objects.

In this study, when invaders (including sympatric species and non-living objects) approached *H. armiger*, the bats would first emit echolocation calls. Then, *H. armiger* would produce different territorial calls in response to the intrusion. We believe that the echolocation calls were mainly used to recognize the invading objects, for the following reasons. First, many studies have confirmed that bats can distinguish the shape and texture of objects through echolocation calls [38,39,40]. Second, bats do not produce odors during approach via flight [41,42], and non-living objects were wiped with alcohol before the experiments. In this case, the odor could not be used as a recognition cue. Third, the experiments were carried out in complete darkness, which suggests that *H. armiger* cannot use visual cues (such as size or shape) to identify invading objects. Therefore, it is likely that *H. armiger* produced echolocation calls to recognize sympatric species and non-living objects, providing the information necessary for bats to respond to the intruders through different territorial calls. Through this process, echolocation calls can play a vital role in addition to territorial calls for *H. armiger* territorial defense.

Motivation-structural rules state that birds and mammals use low-frequency and noisy vocalizations in aggressive or hostile contexts, whereas high-frequency and tonal vocalizations are used in fearful, friendly, or gentle contexts [43]. In our study, *H. armiger* emitted high-frequency calls (64.62 ± 2.7 kHz in peak frequency) and tonal calls to defend roost territories, which may imply that *H. armiger* were in a fearful, friendly, or gentle context. Our previous study has shown that social calls of *H. armiger* encode individual identity and emotional state information during agonistic interactions [44]. Therefore, our results suggest that territorial calls of *H. armiger* may convey emotional state information when the bats respond to intrusions into their territories. However, only by determining a direct physiological basis for territorial calls can this question be fully resolved.

## 5. Conclusions

To our knowledge, our study has provided the first behavioral evidence that *H. armiger* can adjust its territorial calls in response to different sympatric species and non-living objects. *H. armiger* may advertise emotional state information such as fear or gentleness via territorial calls with high-frequency and tonal structure. A limitation of this study was that the physiological state (e.g., body temperature or heart rate) was not monitored when bats responded to the sympatric species and non-living objects. It will be necessary to integrate acoustic behavior and physiological indicators to determine the information contained in territorial calls of *H. armiger* in future studies.

## Supplementary Materials

The following are available online at https://www.mdpi.com/2076-2615/10/11/2040/s1, Figure S1: The territorial calls syllabic types of Hipposideros armiger, Table S1: Definitions of acoustic parameters.
