# Supplementary material for: Great Himalayan Leaf-Nosed Bats Produce Different Territorial Calls to Respond to Sympatric Species and Non-Living Objects
Source: Animals (Basel). 2020 Nov 4;10(11):2040. doi: 10.3390/ani10112040 (PMC7694401; doi:10.3390/ani10112040)
Supplement: Supplementary file 1 [file animals-10-02040-s001.pdf]

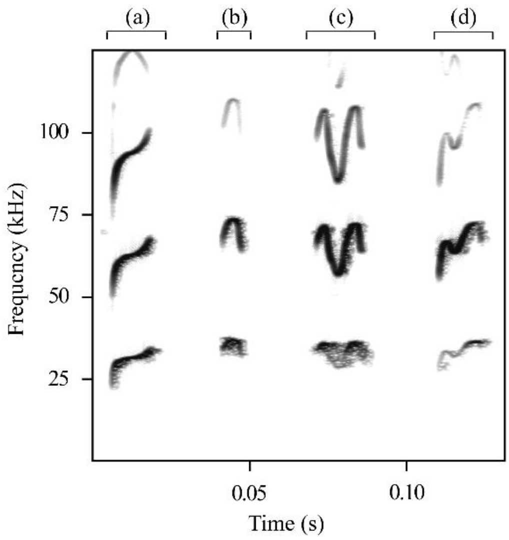

Figure S1. The syllabic types of territorial calls in *Hipposideros armiger*. (a) sUFM (stepped upward frequency modulation). (b) sHFM (single humped frequency modulation). (c) pPFM-sHFM (plateaued paraboloid frequency modulation-single humped frequency modulation). (d) bUFM-sHFM (bent upward frequency modulation-single humped frequency modulation).

Table S1. Definitions of acoustic parameters.

Sound parameter	Definition
Syllable duration(ms)	Time between beginning and end of a syllable
Disttomax	The distance from start to the location of the maximum amplitude
peak freq(start)	Peak frequency at the start location of the syllable
peak ampl(start)	Peak amplitude at the start location of the syllable
min freq(start)	Minimum frequency at the start location of the syllable
max freq(start)	Maximum frequency at the start location of the syllable
bandw(start)	Maximum frequency (start)—Minimum frequency (start)
peak freq(end)	Peak frequency at the end location of the syllable
peak ampl(end)	Peak amplitude at the end location of the syllable
min freq(end)	Minimum frequency at the end location of the syllable
max freq(end)	Maximum frequency at the end location of the syllable
bandw(end)	Maximum frequency (end)—Minimum frequency (end)
peak freq(centre)	Peak frequency at the centre location of the syllable
peak ampl(centre)	Peak amplitude at the centre location of the syllable
min freq(centre)	Minimum frequency at the centre location of the syllable
max freq(centre)	Maximum frequency at the centre location of the syllable
bandw(centre)	Maximum frequency (centre)—Minimum frequency (centre)
peak freq(max)	Peak frequency at the maximum amplitude location of the syllable
peak ampl(max)	Peak amplitude at the maximum amplitude location of the syllable
min freq(max)	Minimum frequency at the maximum amplitude location of the syllable
max freq(max)	Maximum frequency at the maximum amplitude location of the syllable
bandw(max)	Maximum frequency (max)—Minimum frequency (max)
peak freq	Peak frequency at the maximum spectrum location of the entire syllable
peak ampl	Peak amplitude at the maximum spectrum location of the entire syllable
min freq	Minimum frequency at the maximum spectrum location of the entire syllable
max freq	Maximum frequency at the maximum spectrum location of the entire syllable
Bandw	Maximum frequency (maxpeakhold)—Minimum frequency (maxpeakhold)

Quartile 25%	Frequency at the upper limit of the first quartile of energy
Quartile 50%	Frequency at the upper limit of the second quartile of energy
Quartile 75%	Frequency at the upper limit of the third quartile of energy
